# Use of low-cost virtual reality in the treatment of the upper extremity in chronic stroke: a randomized clinical trial

**DOI:** 10.1186/s12984-024-01303-2

**Published:** 2024-01-22

**Authors:** Ángela Aguilera-Rubio, Isabel M. Alguacil-Diego, Ana Mallo-López, Alberto Jardón Huete, Edwin D. Oña, Alicia Cuesta-Gómez

**Affiliations:** 1 Department of Physiotherapy, HM Hospitals Faculty of Health Sciences of the Camilo José Cela University, 28692 Villanueva de la Cañada, Madrid, Spain; 2 Department of Physical Therapy, Occupational Therapy, Rehabilitation and Physical Medicine. Faculty of Health Sciences, Rey Juan Carlos University, Avenida de Atenas S/N, Alcorcón, 28922 Madrid, Spain; 3 Department of Physiotherapy, Faculty of Sport Sciences, Universidad Europea de Madrid, Villaviciosa de Odón, 28670 Madrid, Spain; 4 Systems and Automatics Department, Universidad Carlos III de Madrid, Madrid, Spain

**Keywords:** Leap Motion Controller^®^, Neurorehabilitation, Stroke, Upper limb, Video games, Virtual reality

## Abstract

**Background:**

Chronicity and lack of motivation often go together during the upper limb rehabilitation process in stroke. Virtual reality is a useful tool in this context, providing safe, intensive, individualised treatments in a playful environment. B-cost, easy-to-use devices with personalised and motivating games for a specific population seem to be the most effective option in the treatment of the upper limbs.

**Methods:**

A randomised clinical study with follow-up was carried out to assess the effectiveness of the Leap Motion Controller® device in improving the functionality of the upper limb in patients with chronic stroke. Patients (n = 36) were randomised into a control group that performed conventional therapy and an experimental group that combined the virtual reality protocol with conventional therapy. The outcome measures used were grip strength; the Block and Box Test; the Action Research Arm Test; the Disabilities of the Arm, Shoulder and Hand; as well as a Technology Satisfaction Questionnaire and adherence to treatment.

**Results:**

Inter-group statistical analysis showed no significant differences except in subsection D of the Action Research Arm Test. Intra-group analysis showed significant differences in both groups, but the experimental group reached significance in all long-term variables. Satisfaction and adherence levels were very high.

**Conclusions:**

The Leap Motion Controller^®^ system, as a complementary tool, produces improvements in grip strength, dexterity and motor function in patients with chronic stroke. It is perceived as a safe, motivating, and easy-to-use device.

*Clinical Registration*: NCT04166617 Clinical Trials.

## Introduction

The chronicity of the upper limb (UL) rehabilitation process in stroke leads to an inherent loss of motivation in the long term, resulting in the need for new treatment approaches that combine both the recovery of functionality and the motivation to continue to achieve functional goals [1, 2].

The use of virtual reality (VR) in neurorehabilitation arose with the aim of creating new, more efficient options for functional recovery by generating environments in which to carry out different tasks and is currently a potential treatment tool [3]. VR is a promising tool in neurorehabilitation, as it promotes neuroplasticity by enabling treatments with a high number of repetitions, allowing changes in task difficulty, and keeping patients motivated and involved during the rehabilitation session [4, 5].

The development of portable and affordable VR devices is making VR therapy accessible to the chronic population [6]. A recent review on the design of serious games in the field of neurorehabilitation [7] outlines three necessary concepts when developing this technology: the game genre, the nature of the game, and the game development strategy. The authors concluded that casual games (any game that involves the completion of single, simple tasks, not tied to a story or extensive development) designed specifically for one type of patient, with a first-person perspective, played in single-player mode, and using non-immersive VR had better clinical outcomes. In contrast to this, they also stated that commercial games are perceived as more engaging and motivating, emphasising the need for future work in this area. Therefore, perhaps the trend should be to use inexpensive, easy-to-use devices, but with games that are personalised and motivating for a specific population.

The Leap Motion Controller^®^ (LMC^®^) system is a small, portable, low-cost, and commercially available tracking device that can capture UL movements in 3D, without the need for motion markers. It is a part of semi-immersive VR and allows the development of customised applications thanks to its software development kit [8, 9]. Its use has been investigated both as a single therapy and in combination with conventional treatment in stroke patients, mainly in the acute and subacute phases. In both cases, the ultimate goal has been to assess its effectiveness on parameters of grip strength, dexterity, or motor function [10]. After a review of the current literature on the use of this device in the stroke population [10], we deduce that studies with a larger sample size, with higher methodological quality, and with follow-up evaluations are needed to assess its effectiveness with greater precision.

Prior to this research, a pilot study of the feasibility of the device was developed together with a VR protocol with games created ad hoc for the chronic stroke population [11]. The results were very positive, which generated the need to verify these results with a study of higher methodological quality.

Despite the amount of scientific literature on the use of virtual reality in the field of neurorehabilitation, and the constant changes in devices, there is a lack of clear detailed protocols, reproducible through low-cost devices.

Therefore, the aim of the present research was to assess the effectiveness of the LMC^®^ system through games designed specifically for chronic stroke patients, and whether the potential benefit, if any, would be sustained over time. A semi-immersive VR protocol was developed as an adjunct to conventional rehabilitation treatment in improving the functionality of the UL compared to a conventional treatment group, in addition to assessing motivation and adherence to treatment.

## Methods

### Design

A single-blind, randomised clinical trial (RCT) with follow-up, using non-probability sampling of consecutive cases, is presented. To ensure the methodological quality of the study, the CONsolidated Standards Of Reporting Trials (CONSORT) checklist was followed [12]. The present work was also registered in Clinical Trials (NCT04166617).

### Patients

After selection and acceptance to take part in the study, patients were randomly divided into two groups, the control group (CG) and the experimental group (EG). Randomisation was carried out by blinded selection of a ballot paper inside an envelope by the patients. A ballot marked with an X meant the patient belonged to the EG, and a blank ballot meant they belonged to the CG. Inclusion criteria were a confirmed diagnosis of chronic stroke (more than 6 months of evolution); people over 18 years of age with no upper age limit; subjects of both sexes; being able to sit independently, without posterior support; and having a score on the Fugl-Meyer scale of the upper extremity equal to or higher than 16. Exclusion criteria were additional diagnosis of other pathologies limiting occupational performance, Mini-Mental test score of less than 24, sensory aphasia, visual disturbances not correctable with ocular devices, and a history of epilepsy due to the use of video games.

### Procedure

The protocol of the present study was approved by the local ethics committee (1603201806018). In addition, the ethical principles for medical research in humans of the Declaration of Helsinki and subsequent revisions were followed. Each participant signed the informed consent form after receiving a detailed explanation of the study objectives and the procedures to be used.

**Fig. 1:**
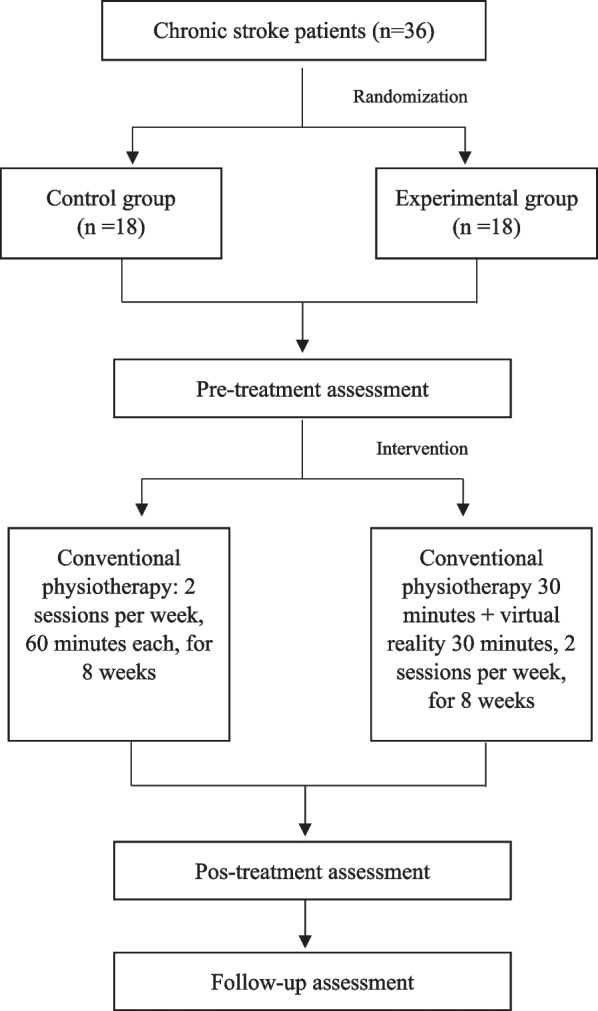
Procedure

The entire sample received two treatment sessions per week of 60 min’ duration for 8 weeks, resulting in a total of 16 sessions. Figure 1 represents the protocol carried out during the present investigation.

The two groups underwent three assessments, all carried out by the same examiner, blinded to the allocation group: pre-treatment (at the start of the intervention), post-treatment (at the end of the intervention), and a final follow-up (4 weeks after the end of the intervention). All patients underwent both the assessments and the intervention at their centre of origin between November 2018 and March 2022. All patients were recruited from different private physiotherapy clinics in Madrid.

**Fig. 2:**
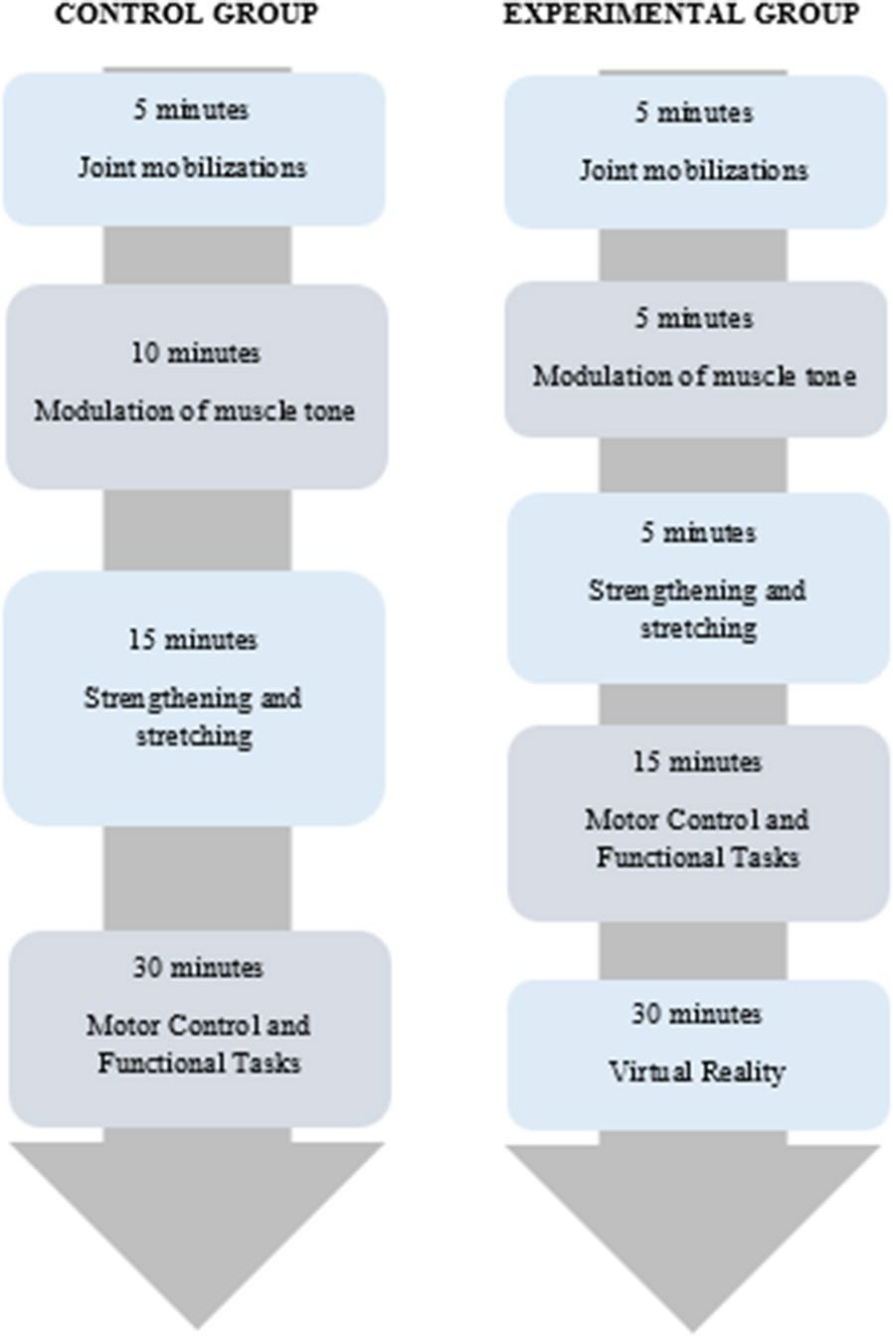
Intervention protocol of the control and experimental groups. Joint mobilizations: shoulder girdle, shoulder, elbow, and wrist; Modulation of muscle tone: stabilizing muscles of the scapula and the entire upper limb; Strengthening and stretching upper limb musculature; Motor Control and Functional Task: tasks focused on activities of daily living such as dressing, eating, or performing household chores

The bulk of each session in the CG was spent on functional tasks, while the EG spent the bulk of each session on the VR intervention (Fig. 2).

The characteristics of each of the games used in the VR protocol are detailed in Table 1. The VR protocol applied was the same as that developed in the previous feasibility pilot study, designed specifically for stroke patients [11]. The virtual environment was created by a multidisciplinary group of clinicians and engineers using the Unity3D Game Engine software. It consists of 4 games, designed specifically for stroke patients. All of them are aimed at improving UL functionality through movements that this population often has altered such as: shoulder girdle stability, shoulder joint movements, elbow flexion–extension, forearm pronation-supination, finger flexion–extension or palmar flexion.

**Table 1:** Characteristics of the serious games used

Serious game	Description
Reach game	In this game, several fruit -shaped objects are shown within the reaching range of the user’s upper extremity represented in different locations. The user has to touch the fruit that is highlighted. Once the fruit is reached, the gravity is activated so it falls to the floor of the virtual scene. To complete the game, the user must reach all cubes showed in random order
Sequence game	This game uses the same set-up as the Reach Game. Now a sequence of fruits is presented to the user, who must memorize the sequence and repeat it by reaching the fruits in the same order shown, adding a cognitive training to the game
Flip game	This game trains pronation and supination movements of the forearm. The user must place the palm of the hand over the LMC^®^ device imitating a waiter holding out a tray. A small tray with a cube in the middle appears in the center of the screen. The patient should then turn the palm downwards. Upon doing so, the cube detaches from the tray and falls to the ground
Opening/closing game	This game encourages the user to achieve the opening and/or closing of the hand, simulating grasping movements, depending on the percentage specifically programmed for each of them. A red circle in the centre of the screen is showed to indicate to the user where to place the fruits into. When a fruit is highlighted, the user must grasp it and move it to the red circle while keeping their hand closed, and keep this gesture until the hand touches the red circle. Then the user has to open the hand

All games had the possibility of starting unilaterally. It was decided to start with the unaffected hand (so that the patient could become familiar with the game), continuing later with the affected hand. Finally, each game was played with both hands simultaneously, except for the game known as "sequence", which did not offer this possibility.

In order to be able to adapt the degree of difficulty to the particular needs of each patient, the games can be customized and individualized. In each of the games, the number of objects that initially appear on the screen, the distances between them and the depth at which they are placed can be configured. All these settings are recorded and stored in each patient's profile. Furthermore the level of difficulty was adjusted, always starting with easier games, and concluding with more difficult games.

During VR therapy, the patient remained seated, with the feet in contact with the floor, and to avoid compensatory movements, the trunk was restricted by means of an elastic strap. Likewise, the hand that was not being used at the time of the game was placed on the table in order to facilitate midline orientation and the increase of axial tone.

### Outcome measures

#### Grip strength

The Jamar hydraulic dynamometer^®^ (Model 5030J1 serial number 20210511237) was used to measure the grip strength of the affected hand, following the recommendations of the American Hand Association [13], and position II of the dynamometer handle was set as the standard size.

#### Block and box test (BBT)

An outcome measure to assess gross manual dexterity. In this study, values were recorded only for the affected hand, although, as the test requires, a previous attempt was made with the unaffected hand [14].

#### Action research arm test (ARAT)

A reliable assessment tool to evaluate the motor function of the UL after stroke. A distinction was made between the four sections (A, B, C, D) of the test, which was maintained when analysing the results [15].

#### Disabilities of the arm, shoulder and hand (DASH)

A self-administered and specific questionnaire to measure the function of the UL. A direct relationship is established between the different parts of the UL and the patient’s perceived difficulty in carrying out certain activities [16].

#### Technology satisfaction questionnaire

The research group designed a specific questionnaire based on a Likert-type scale in order to assess patient satisfaction, since patient satisfaction is a clear additional indicator of the effectiveness of a therapy, as it improves both treatment compliance and adherence to treatment [1, 2, 17]. The questionnaire consisted of 9 items that assessed the usefulness of the LMC^®^ in their rehabilitation, the degree of motivation, possible technical problems during the intervention, usability, possible pain reported during therapy, the importance of therapist support, experience, frequency of use of electronic devices and the use of new technologies in the rehabilitation process. The range of the questionnaire was from 1 to 4, with a maximum possible score of 36 points. All questions were directly proportional, i.e. the higher the score, the better the patient’s perception.

#### Treatment adherence rate

The percentage of attendance was recorded, as was the presence of adverse effects in both treatment groups.

### Statistical analysis

Using G*power software (version 3.1.7), the sample size was calculated, resulting in a minimum of 36 participants for the study. The estimated effect size for the main outcome measures established in this study was 0.30, considering a power of the statistical test of 0.95, an alpha error of 0.05, a correlation between repeated measurements of 0.5 (two groups, three measurements), a sphericity correction coefficient of 1, and a loss rate of 20%.

Statistical analysis was carried out using Statistical Package for the Social Sciences (SPSS) 24.0 statistical software for Windows (SPSS Inc., Chicago, IL, USA; version 24.0). Since the sample followed a normal distribution, repeated measures analysis of variance (ANOVA) was performed for the variables grip strength, BBT, ARAT, and DASH, with time (pre-treatment, post-treatment, follow-up) as an intra-group factor and group (experimental and control) as an inter-group factor, all with Bonferroni post-hoc adjustment. The p-values associated with the ANOVA F-statistics were adjusted using the Greenhouse–Geisser correction. The statistical analysis was performed at a 95% confidence level, so p-values of less than 0.05 were considered significant.

## Results

The sample consisted of 36 patients. The characteristics of the sample are shown in Table 2.

**Table 2:** Characteristics of the sample

	n = 36	Experimental group (n = 18)	Control group (n = 18)	P value
Age	64.94 (± 12.33)	60.33 ± 12.44	69.56 ± 10.63	0.230
Sex				
Male	20 (55.56%) 16 (44.44%)	8 (44.44%)	12 (66.67%)	0.190
Female		10 (55.56%)	6 (33.33%)	
Years since stroke onset	5.78 (± 5.42)	5.64 ± 6.26	5.92 ± 4.60	0.883
Type				
Ischaemic	32 (88.89%) 4 (11.11%)	15 (83.33%)	17 (94.44%)	0.105
Haemorrhagic		3 (16.67%)	1 (5.56%)	
Affected side				
Left	20 (55.56%) 16 (44.44%)	11 (61.11%)	9 (50%)	0.331
Right		7 (38,89%)	9 (50%)	

Data expressed as mean, standard deviation and frequencies (percentages)

Student's t-test for independent samples. *p value < 0.05

*p value statistically significant

Statistical analysis showed no statistically significant inter-group differences except in ARAT subsection D (gross movements), which was significant in the post-treatment assessment (p = 0.044) (Table 3). Intra-group analysis showed significant differences in different variables in both groups. The EG recorded statistically significant changes in grip strength in pre- and post-treatment assessments (p = 0.021) and between pre-treatment and follow-up (p < 0.001); in BBT between pre-treatment and post-treatment (p = 0.001) and between pre-treatment and follow-up (p < 0.001); in the total ARAT assessment between pre- and post-treatment (p = 0.002); in ARAT subsections A (grip) and C (grip) between pre-treatment and follow-up (p = 0.029 and p = 0.009, respectively); and finally, in the DASH questionnaire between pre- and post-treatment assessments (p = 0.004) and between pre-treatment and follow-up (p = 0.007). The CG obtained the following significant differences: in the BBT between pre-treatment and follow-up assessment (p = 0.014) and between post-treatment and follow-up assessments (p = 0.005), and in the DASH questionnaire in the pre-treatment versus follow-up assessment (p = 0.035) (Table 4).

**Table 3:** Inter-group results

Variable	EG	CG	F	P	Bonferroni		
	Media ± SD	Media ± SD			MD	P	CI
Grip strength pre	14.87 ± 8.07	21.33 ± 12.71	1.855	0.174	− 6.47	0.077	(− 13.68 to 0.74)
Grip strength pos	16.92 ± 8.29	22.63 ± 12.43			− 5.71	0.114	(− 12.87 to 1.44)
Grip strength f-up	17.61 ± 9.08	22.50 ± 12.58			− 4.89	0.190	(− 12.32 to 2.54)
BBT pre	23.78 ± 12.42	31.56 ± 11.71	3.046	0.076	− 7.78	0.062	(− 15.95 to 0.40)
BBT pos	27.17 ± 13.27	32.50 ± 10.68			− 5.33	0.193	(− 13.50 to 2.83)
BBT f-up	27.72 ± 12.13	33.89 ± 10.89			− 6.17	0.118	(− 13.96 to 1.64)
ARAT pre	41.78 ± 14.94	48.78 ± 7.52	0.549	0.498	− 7.00	0.085	(− 15.01 to 1.01)
ARAT pos	45.06 ± 14.51	50.17 ± 7.16			− 5.11	0.189	(− 12.86 to 2.64)
ARAT f-up	42.94 ± 16.78	50.56 ± 6.75			− 7.61	0.083	(− 16.27 to 1.05)
ARAT (A) pre	13.39 ± 5.18	16.00 ± 2.47	0.717	0.420	− 2.61	0.062	(− 5.36 to 0.14)
ARAT (A) pos	14.17 ± 4.99	16.50 ± 2.23			− 2.33	0.079	(− 4.95 to 0.28)
ARAT (A) f-up	14.50 ± 4.93	16.50 ± 2.23			− 2.00	0.126	(− 4.59 to 0.59)
ARAT (B) pre	9.78 ± 2.96	11.17 ± 1.47	0.513	0.491	− 1.39	0.083	(− 2.97 to 0.19)
ARAT (B) pos	10.28 ± 2.56	11.39 ± 1.24			− 1.11	0.107	(− 2.47 to 0.25)
ARAT (B) f-up	10.39 ± 2.55	11.44 ± 1.20			− 1.06	0.664	(− 2.40 to 0.29)
ARAT (C) pre	10.72 ± 6.27	12.83 ± 4.84	1.647	0.209	− 2.11	0.266	(− 5.90 to 1.68)
ARAT (C) pos	12.44 ± 6.39	13.28 ± 4.90			− 0.83	0.663	(− 4.69 to 3.02)
ARAT (C) f-up	12.56 ± 6.30	13.61 ± 4.43			− 1.06	0.565	(− 4.74 to 2.63)
ARAT (D) pre	7.83 ± 1.79	8.78 ± 0.94	0.375	0.572	− 0.94	0.056	(− 1.91 to 0.03)
ARAT (D) pos	8.17 ± 1.69	8.98 ± 0.02			− 0.83	0.044*	(− 1.64 to − 0.02)
ARAT (D) f-up	8.28 ± 1.65	8.98 ± 0.02			− 0.72	0.076	(− 0.08 to 1.52)
DASH pre	42.98 ± 16.07	37.22 ± 19.23	0.382	0.564	5.78	0.335	(− 6.23 to 17.79)
DASH pos	40.35 ± 16.22	35.35 ± 17.23			4.98	0.377	(− 6.34 to 16.33)
DASH f-up	40.45 ± 15.68	35.14 ± 17.28			5.31	0.341	(− 5.86 to 16.49)

*EG* experimental group, *CG* control group, *SD* standard deviation, *MD* mean difference, *CI* confidence interval, *Pre* pre-treatment, *Pos* pos-treatment, *F-up* follow-up, *BBT* block and box test, *ARAT* action research arm test, *ARAT (A)* action research arm test subtest A grasp, *ARAT (B)* action research arm test subtest B grip, *ARAT (C)* action research arm test subtest C pinch, *ARAT (D)* action research arm test subtest D gross movement, *DASH* disabilities of the arm, shoulder and hand questionnaire

Repeated measures analysis of variance (ANOVA) with Bonferroni a posteriori adjustment. *p value < 0.05

**Table 4:** Intra-group results

Variable	G	Media ± SD			F	P	Pre vs Pos			Pre vs f-up			Pos vs f-up		
		Pre	Pos	f-up			MD	P	CI	MD	P	IC	MD	P	CI
Grip Strength	EG	14.87 ± 8.07	16.92 ± 8.29	17.61 ± 9.08	13.278	< 0.001*	− 2.06	0.021*	(− 3.86 to − 0.25)	− 2.74	< 0.001*	(− 4.12 to − 1.37)	− 0.69	0.393	(− 1.81 to 0.43)
	CG	21.33 ± 12.71	22.63 ± 12.43	22.50 ± 12.58			− 1.30	0.237	(− 3.10 to 0.51)	− 1.16	0.122	(− 2.54 to 0.21)	0.132	1.000	(− 0.99 to 1.25)
BBT	EG	23.78 ± 12.42	27.17 ± 13.27	27.72 ± 12.13	20.370	< 0.001*	− 3.39	0.001*	(− 5.58 to − 1.20)	− 3.94	< 0.001*	(− 5.89 to − 2.00)	− 0.56	0.552	(− 1.59 to 0.48)
	CG	31.56 ± 11.71	32.50 ± 10.68	33.89 ± 10.89			− 0.94	0.861	(− 3.14 to 1.25)	− 2.33	0.014*	(− 4.28 to − 0.39)	− 1.39	0.005*	(− 2.42 to − 0.36)
ARAT	EG	41.78 ± 14.94	45.06 ± 14.51	42.94 ± 16.78	1.798	0.187	− 3.28	0.002*	(− 5.43 to − 1.12)	− 1.17	1.000	(− 6.72 to 4.38)	2.11	0.841	(− 2.74 to 6.96)
	CG	48.78 ± 7.52	50.17 ± 7.16	50.56 ± 6.75			− 1.39	0.341	(− 3.54 to 0.77)	− 1.78	1.000	(− 7.33 to 3.77)	− 0.39	1.000	(− 5.24 to 4.46)
ARAT (A)	EG	13.59 ± 5.18	14.17 ± 4.99	14.50 ± 4.93	5.535	0.020*	− 0.78	0.293	(− 1.93 to 0.37)	− 1.11	0.029*	(− 2.13 to − 0.92)	− 0.33	0.069	(− 0.69 to 0.19)
	CG	16.00 ± 2.47	16.50 ± 2.23	16.50 ± 2.23			− 5.00	0.84	(− 1.65 to 0.65)	− 5.00	0.68	(− 1.52 to 0.51)	0.00	1.00	(− 0.35 to 0.35)
ARAT (B)	EG	9.78 ± 2.96	10.28 ± 2.56	10.39 ± 2.55	3.589	0.033*	− 0.50	0.337	(1.27 to 0.27)	− 0.61	0.142	(− 1.36 to 0.14)	− 0.11	0.315	(− 0.28 to 0.06)
	CG	11.17 ± 1.47	11.39 ± 1.24	11.44 ± 1.20			− 0.22	1.000	(− 0.99 to 0.55)	− 0.28	1.000	(− 1.03 to 0.47)	− 0.06	1.000	(− 0.22 to 0.11)
ARAT (C)	EG	10.72 ± 6.27	12.44 ± 6.39	12.56 ± 6.30	6.898	0.010*	− 1.72	0.051	(− 3.45 to 0.01)	− 1.83	0.009*	(− 3.28 to − 0.38)	− 0.11	1.000	(− 0.65 to 0.43)
	CG	12.83 ± 4.84	13.28 ± 4.90	13.61 ± 4.43			− 0.44	1.000	(− 2.17 to 1.28)	− 0.78	0.559	(− 2.23 to 0.68)	− 0.33	0.381	(− 0.87 to 0.20)
ARAT (D)	EG	7.83 ± 1.79	8.17 ± 1.69	8.28 ± 1.65	3.875	0.051	− 0.33	0.289	(− 0.82 to 0.16)	− 0.44	0.199	(− 1.03 to 0.15)	− 0.11	0.499	(− 0.31 to 0.09)
	CG	8.78 ± 0.94	8.98 ± 0.02	8.98 ± 0.02			− 0.22	0.782	(− 0.71 to 0.27)	− 0.22	1.000	(− 0.81 to 0.37)	0.00	1.000	(− 0.20 to 0.20)
DASH	EG	42.98 ± 16.07	40.35 ± 16.22	40.45 ± 15.68	17.20	< 0.001*	2.65	0.004*	(0.76 to 4.54)	2.55	0.007*	(0.59 to 4.51)	− 0.10	1.000	(− 0.65 to 0.44)
	CG	37.22 ± 19.23	35.35 ± 17.23	35.14 ± 17.28			1.87	0.053	(− 0.02 to 3.76)	2.08	0.035*	(0.12 to 4.05)	0.21	0.998	(− 0.34 to 0.76)

*G* group, *EG* experimental group, *CG* control group, *SD* standard deviation, *MD* mean difference, *CI* confidence interval, *Pre* pre-treatment, *Pos* pos-treatment, *F-up* follow-up, *BBT* Block and Box Test, *ARAT* Action Research Arm Test, *ARAT (A)* Action Research Arm Test Subtest A grasp, *ARAT (B)* Action Research Arm Test Subtest B grip, *ARAT (C)* Action Research Arm Test Subtest C pinch, *ARAT (D)* Action Research Arm Test Subtest D gross movement, *DASH* Disabilities of the Arm, Shoulder and Hand Questionnaire

Repeated measures analysis of variance (ANOVA) with Bonferroni a posteriori adjustment. *p value < 0.05

With respect to the satisfaction questionnaire related to the VR protocol performed by the EG, the degree of satisfaction was satisfactory. The mean score obtained was 29.83 (± 2.792), out of a maximum score of 36 (Table 5).

**Table 5:** Results of the technology satisfaction questionnaire for the experimental group

Ìtem	Media—SD
Motivation	3.56 (0.616)
Usability	3.00 (0.767)
Use of electronic devices	2.78 (0.877)
Pain	3.61 (0.502)
Therapist´s help	3.83 (0.383)
Technical problems	2.56 (0.856)
Utility	3.39 (0.608)
Experience	3.28 (0.574)
New technologies	3.83 (0.383)
Total score	29.83 (2.792)

Data expressed as mean and standard deviation (SD)

With regard to adherence to treatment, the CG completed an average of 15.67 sessions, representing 97.93%, and the EG completed the intervention with an average of 15.78 sessions, i.e. 98.63%.

Also, no patients dropped out of the trial and there were no adverse effects other than occasional mild shoulder discomfort at the end of the treatment session in some participants (n = 4).

## Discussion

The purpose of the present study was to assess the effectiveness of the LMC^®^ system in a semi-immersive VR protocol, as an adjunct to conventional rehabilitation treatment, in improving the functionality of the UL through grip strength, dexterity, and motor function compared to a conventional treatment group. In addition, the degree of satisfaction with the device was assessed, as well as adherence to treatment.

To our knowledge, the present investigation is the first RCT with follow-up to assess the effectiveness of the LMC^®^ device in improving the functionality of the UL, through grip strength, dexterity, and motor function, in patients with stroke in the chronic phase. Satisfaction with the device and the rate of adherence to treatment were also considered.

Regarding the VR protocols with the LMC^®^ device observed in the literature, each author handled different times, numbers of sessions, and frequencies [10]. The time spent in VR ranged from 15 to 45 min [11, 18–23], in interventions where it was supplemented with conventional therapy, the total treatment session time ranged from 45 to 60 min [11, 18–21] or even 90 [23] or 110 [22] minutes. In reference to the number of sessions, Aramaki et al. [29] in their review on VR in the rehabilitation of stroke patients, highlight the predominance of protocols of short and moderate duration, in addition to low intensity in terms of the number of weekly sessions. The intervention methodology of most of the studies analysed in this review was to apply two sessions per week, lasting between 30 and 60 min each, for a period of 6 weeks. The total number of sessions also varied, although most studies conducted between 9 and 20 sessions, spread over 4–12 weeks [10, 11]. In summary, the amount of therapy with the LMC^®^ device does not seem to be a determining factor in the improvement of the functionality of the UL, as significant improvements have been achieved with different times, numbers of sessions, and frequencies [10, 11].

Follow-up assessment usually varies in the same way in the literature, but our work coincides with that proposed in the review by Jin et al. [4], where the 4-week period is established as the most widely adopted in VR studies in patients with UL impairment after stroke.

With regard to grip strength, recovery is the primary goal. Muscle weakness is the most common impairment of the UL after stroke [24], leading, in addition to the impact on activities of daily living (ADLs), to potential long-term problems such as decreased bone density and thus risk of fracture [25]. All authors who assessed it after intervention with the LMC^®^ found significant improvements. Vanbellingen et al. [19] improved their participants’ grip strength by 11.3% in a single VR intervention. Similarly, but in combination with conventional therapy, improvements in grip strength were also achieved [11, 22]. Iosa et al. [22] attribute them to the fact that the LMC^®^ system allows the capture and reproduction of movements with all degrees of freedom at the wrist and fingers. In our case, we obtained improvements in both groups, and the GE achieved significance between pre- and post-treatment assessments, and between pre-treatment and follow-up. Although the VR protocol did not include any specific muscle-strengthening exercises, the GE achieved significance. Improved stabilisation of the shoulder girdle in stroke patients is a necessary precursor to improved grip strength [26]. Following this premise, our results on improvements in gross movements (ARAT D) could justify improvements in grip strength.

Our results on gross motor dexterity assessed by the BBT show that both groups achieved the minimum detectable change necessary (1.99 blocks) to translate into clinical improvements in the affected hand [27]. The BBT was also the test of choice for other authors [11, 21] to measure gross manual dexterity, in both cases showing significance. In contrast, two other studies opted for the Nine Hole Peg Test as a method for assessing fine dexterity. Iosa et al. [22] did not obtain significance, although they did obtain improvements, while Vanbellingen et al. [19] did achieve significant differences. It should be noted that, in these two studies, some of the games developed involved selective finger movements, unlike those developed for our study.

In relation to motor function assessed by ARAT**,** there is strong scientific evidence of its improvement after VR protocols in stroke patients. It is one of the aspects most frequently addressed in research on VR, and there are different tools for its assessment [28, 29]. In the present investigation, despite the improvements in all subgroups and the significance obtained, no minimal clinically important differences were achieved in either treatment group. A score of 5.7 has been established as the value that determines these minimal differences [18], and in our case, all improvements were below this figure. Two papers [11, 18] used this test in their research on LMC^®^ and stroke, in both cases showing significant differences. On the other hand, in one of them [18], in contrast to our results, there were minimal clinically important differences in EG, perhaps because their protocol was more intensive (three sessions per week for 6 weeks, using VR as the only therapy), and/or their intervention was immersive, as it combined the LMC^®^ with Oculus Rift^®^ (Oculus VR, USA) goggles. However, the authors did not break down their results into subgroups by differentiating between different motor functions. In order to gather more information about the UL motor function of the participants, we decided to include the DASH questionnaire. Although we relied on the patient’s subjective opinion, it allowed us to measure and quantify the degree of difficulty in performing certain ADLs. Despite being a validated questionnaire in stroke patients, its use is not very widespread in relation to VR, as we have seen both in different systematic reviews [3, 4, 28–31] as well as in articles where LMC^®^ was used in relation to stroke [11, 18–23].

In this investigation, it was decided not to measure participants' spasticity, since objective and validated outcome measures are not available to all clinicians. Although the Modified Ashworth Scale is the gold standard manual tool for assessing spasticity, there is controversy as to whether it should remain so [32], given its enormous inherent subjectivity component. Resistance to passive movement is not solely due to reflex muscle activity, but is also influenced by non-neural mechanical characteristics. In chronic patients, it is common that the viscoelastic properties of joint structures and soft tissues are frequently altered. Some authors propose the use of this scale in conjunction with instrumented monitoring systems or electromyography [32], while other authors opt for instrumental systems alone [33]. The aim would be to be able to accurately discriminate these components, to understand their influence on motor recovery and to optimize treatment options on an individual basis. Perhaps this argument justifies why spasticity is not a variable that is overly analysed in VR studies in the chronic patient.

The patient’s opinion is fundamental in any neurorehabilitation process, and in our research, we used an ad-hoc Technology Satisfaction Questionnaire. Patient satisfaction in these processes has an inherent relationship with adherence to treatment and therefore with the quality of life of patients. It is common for people who have suffered a stroke, and are in chronic phases, not to maintain the motivation to go to rehabilitation for long periods of time. Considering that in many cases the treatment is carried out throughout life (in lesser or greater amounts), we consider that it is important to know the individual experience after the use of new tools. In this way, more individualized intervention models can be adjusted [34].

“Technical problems during the intervention” was the item with the lowest score (2.56). In particular, the lack of a signal from the device was a recurrent complaint from patients. This is in line with Iosa et al. [22], who attribute it to the LMC^®^ sensors’ lack of ability to follow the movements of the fingers when the hands are overlapped, or when there is a high level of spasticity. This same problem has also been reported in a previous study [11]. The next item with the lowest score (2.78) was “the use of electronic devices”, from which we wanted to learn the frequency with which participants used either mobile phones, computers, or video game consoles. Although the mean age of the EG was 60.33 years, we associated that the older the age, the lower the frequency of use of this type of device and, consequently, the greater the difficulty of use. However, this question was addressed by another article [22] in which the LMC^®^ device was applied in older patients in the subacute phase, without the sample showing any problems with its use, although the participants’ satisfaction was not assessed.

On the other hand, the items with the highest scores were “the incorporation of new technologies into the rehabilitation process” and “the therapist’s help during the intervention”, especially during the first treatment sessions, both with a score of 3.83 points out of 4 [30, 31]. The help of a therapist during the play period also appeared in other similar studies [11, 19, 22, 23], concluding, as in our work, the advantage of having a clinician nearby at the beginning of the intervention.

Pain was another of the questionnaire items with the best score (3.61). Only a few patients reported occasional mild shoulder discomfort at the end of the treatment session, compatible with tendon overloads, which disappeared after the use of the therapy, and which at no time made it necessary to interrupt it. Nor was there any other type of discomfort, since, as indicated by Ogün et al. [18], the new generation of VR devices avoid symptoms such as dizziness, nausea, and headache, which is their biggest advantage compared to older devices. However, as no specific pain scale was used, the data provided by the questionnaire cannot be considered conclusive regarding pain.

Stroke, like the vast majority of chronic neurological diseases, given the care resources available, does not allow these patients to be treated continuously over time, and they are discharged from rehabilitation services after around 6 months [35, 36]. Current evidence has shown that improvements in functionality are still possible in the chronic patient [36]. This should force a change in the paradigm of care for these patients, but until this happens, and in the face of limited social and health care resources, the implementation of this type of treatment tool must be considered.

### Limitations

The present study is not without limitations. The main limitation was the lack of stratification of patients in relation to their level of involvement. This would have allowed a more accurate assessment of the results, given the clinical heterogeneity inherent to UL involvement in stroke, as it is a way of homogenising the sample. For this same reason, together with the need for a larger sample size, our results cannot be extrapolated to the entire chronic stroke population. Furthermore, the differences in some of the variables in the initial evaluations mean that the results should be taken with caution.

Would have been interesting to measure the baseline spasticity of the sample, and perhaps it can be included in future studies, but with objective instrumental tools.

Although common guidelines and directives were given, the therapy was applied by different therapists, and therefore the patients’ different backgrounds are a limitation.

To determine the full effectiveness of the LMC^®^ as a therapeutic tool for chronic stroke patients, other comparisons, such as conventional treatment versus VR protocol as a single intervention or comparison with other types of VR devices, were not considered.

## Conclusions

The LMC^®^ system produces improvements in the gross motor skills of chronic stroke patients in combination with conventional therapy treatment versus conventional physiotherapy. As a complementary tool, it produces improvements in grip strength, dexterity, and motor function. Similarly, the LMC^®^ system is perceived as a safe, motivating, and easy-to-use tool and provides a very high level of adherence.

## Data Availability

All the data and materials could be found at Faculty of Health Sciences of Rey Juan Carlos University.

## Abbreviations

UL: Upper limb
VR: Virtual reality
LMC®: Leap Motion Controller^®^
BBT: Block and box test
ARAT: Action research arm test
DASH: Disabilities of the arm, shoulder and hand questionnaire
RCT: Randomised clinical trial
CG: Control group
EG: Experimental group
